# The sensitivity of a lower limb model to axial rotation offsets and muscle bounds at the knee

**DOI:** 10.1177/0954411912439284

**Published:** 2012-09

**Authors:** Dominic FL Southgate, Daniel J Cleather, Robert A Weinert-Aplin, Anthony MJ Bull

**Affiliations:** 1 Bioengineering Department, Imperial College London, UK; 2 St. Mary’s University College, UK

**Keywords:** Musculoskeletal modelling, optimisation, knee forces, shank rotation

## Abstract

Soft tissue artifacts during motion capture can lead to errors in kinematics and incorrect estimation of joint angles and segment motion. The aim of this study was to evaluate the effect of shank segment axial rotation and knee rotator muscle bounds on predicted muscle and joint forces in a musculoskeletal model of the lower limb. A maximal height jump for ten subjects was analysed using the original motion data and then modified for different levels of internal and external rotation, and with the upper force bound doubled for five muscles. Both externally rotating the shank and doubling the muscle bounds increased the ability of the model to find a solution in regions of high loading. Muscle force levels in popliteus and tensor fascia latae showed statistically significant differences, but less so in plantaris, sartorius or gracilis. The shear and patellofemoral joint forces were found to be significantly affected by axial rotation during specific phases of the motion and were dependent on the amount of rotation. Fewer differences were observed when doubling the muscle bounds, except for the patellofemoral force and plantaris and sartorius muscle force, which were significantly increased in many of the jump phases. These results give an insight into the behaviour of the model and give an indication of the importance of accurate kinematics and subject-specific geometry.

## Introduction

There are many applications for musculoskeletal models that estimate the muscle and joint forces in the lower limb; for example, as a research tool, for clinical use or for sports performance assessment. However, in order for a model to produce meaningful results the inputs must accurately represent the motion being tested. There has been much interest recently in quantifying soft tissue artifacts during motion analysis and investigating their effect on the calculated kinematics of the lower limb.^1–4^ Very few studies though have extended this analysis to look at what impact these errors might have on predicted muscle and joint loading. Previous investigations using the lower limb model employed in this study^5,6^ have indicated that these loads might be particularly sensitive to non-sagittal plane (non-flexion) rotations at the knee and the muscles that control these motions.

The starting point for most inverse dynamics models is kinematic data recorded for an individual and this is one stage where errors may be introduced that could have an effect later in the modelling sequence. It is possible that local segment co-ordinate frames (such as the shank or thigh) could be constructed incorrectly owing to poor initial digitisation of landmarks. Another source of inaccuracies in motion capture may be marker position errors owing to soft tissue artifacts. Either of these issues could give rise to rotational errors in the limb segments. Of the two non-sagittal plane rotations at the knee, internal/external rotation and varus/valgus, the former is more likely to give greater errors, as the latter would require the markers, or digitised landmarks at the ankle, to move radially, rather than circumferentially relative to the segment.

Modern musculoskeletal models with many muscle elements have a high degree of redundancy and as such, can often have many possible solutions to the equations of motion. However, as constraints are introduced into the model, the number of possible solutions begins to diminish and it can be that the model is unable to find a viable combination of muscle forces. This can be a particular issue for highly dynamic activities that involve fast movements and/or large external loads.^5^ One modelling parameter that can affect the ability of a model to find a solution is the upper bound of the muscle force. This is normally specified as the product of the maximum permitted muscle stress and the physiological cross-sectional area (PCSA), which varies for each muscle. Increasing the PCSA for all the muscles allows each of them to produce a higher force and a viable model solution is often found with this extra capacity added (i.e. a solution where the optimisation can find a set of muscle forces that satisfy all the constraints of the force-sharing problem). However, in many cases the increased upper bound is only required in a small number of key muscles particular to that motion and other muscle and joint forces can actually be reduced as a result.

The aim of the kinematic sensitivity section of this study was, therefore, to use a lower limb musculoskeletal model to investigate the effect of shank segment internal/external rotation on the joint reaction and muscle forces at the knee. The aim of the knee rotator muscle sensitivity analysis was to investigate the effect of altering the upper force bounds of these particular muscles in the optimisation stage of the model.

## Materials and methods

### The lower limb model

The three-dimensional (3D) musculoskeletal model of the right lower limb used in this study has been described in detail previously.^5–10^ To summarise, the model consists of four rigid segments; the foot, shank, thigh and pelvis, connected by ball and socket (three degree of freedom) joints at the ankle, knee and hip. The geometry of the model was based on the cadaveric data of Klein Horsman et al.,^11^ with linear scaling factors for each subject in this study based on a comparison of the anthropometry for their specimen. The segment inertial parameters for the model were scaled based on body mass and height using the anthropometric model of de Leva.^12^ The model uses motion data, as well as ground reaction vector (GRV) and centre of pressure (CoP) data, as input to a one-step method for calculating the inverse dynamics forces and moments in 3D. This method uses unit quaternions and the wrench notation described by Dumas et al.,^13^ and verified in a previous study.^8^

The optimisation stage estimates the muscle forces based on a criterion that aims to minimise the sum of the muscle stresses to power, n. This cost function was based upon the work of Crowninshield and Brand,^14^ who suggested that an optimal force sharing (based on maximising muscular endurance) could be achieved by using this criterion. However, contrary to the recommendations of the aforementioned article (who suggest n = 2–4) a value of n = 30 was employed in this article. This is consistent with the suggestion that as n is increased the solution will tend to a limit that is equivalent to the solution of the min–max criterion^15,16^ and that ensures a more physiological recruitment of the musculature with increasing load.^16^ This criterion forces a more even recruitment of the musculature that the authors believe is more consistent with the imperative for maximising muscular endurance and has been used in the previous studies employing this model.^5,7–10^

The model calculates the resultant tibiofemoral joint contact force and patellofemoral joint contact force once the muscle forces have been estimated. The resultant anterior–posterior shear force in the knee is assumed to be resisted by the cruciate ligaments, where the absolute value in the recruited ligament is dependent on the angle of flexion. The resultant medio-lateral shear force is assumed to be resisted by the collateral and ancillary ligaments in the knee, however, these ligaments were not discretely modelled in the study.

### The motion dataset

Motion data for 10 males from an athletic population was used in this study (mean age 26.6 ±4.3 y, mean mass 83.5 ±10.9 kg, mean height 1.79 ±0.07 m). Subjects performed five vertical jumps from a static standing position, with the right foot located over a force platform (Kistler Type 9286AA, Kistler Instrumente AG, Winterthur, Switzerland). Motion capture was performed using a Vicon MX system (Vicon Motion Systems Ltd, Oxford, UK) running at 200 Hz. The force platform data was sampled at 200 Hz and both ground reaction force data and marker data were filtered using a fifth-order Woltring filter, with a cut-off frequency of 10 Hz.

The highest of the five jumps was selected for analysis to ensure that it was truly a maximal jump and not skewed by averaging sub-maximal trials (albeit maximal attempts). An indication of the variability in data was instead sought through the testing of multiple subjects. The mean maximum jump height, taken as the vertical distance travelled by the heel marker on the foot, was 532 mm. This was 60 mm higher than the average height of the 5 jumps across all subjects.

The kinematic data was used to reconstruct the position and orientation of each segment using the method of Horn.^17^. The raw marker positions were used to generate the local co-ordinate system (LCS) for each segment and then unit quaternions were created that describe the transformation between each LCS and the global co-ordinate system (GCS). The first and second derivatives of these unit quaternions (velocities and accelerations) were then found so that they could then be used in the inverse dynamics stage.

### Kinematic sensitivity analysis

The original ‘highest jump’ data was processed for each subject using the lower limb model. The unit quaternions that represent the segmental rotation were then modified to simulate four angles of axial rotation of the shank segment relative to the original motion data; +0.1 rad, +0.05 rad, −0.05 rad and −0.1 rad, where internal rotation is positive. These values fall within previous estimates of the peak error in shank rotation about the longitudinal axis, which have been as high as 8° (0.14 rad).^18^ The model was re-run for these modified angles, keeping all other parameters constant.

It should be noted that, while altering the axial rotation of the shank segment could theoretically alter the inverse dynamics, the shank inertial properties were assumed to be symmetrical about the long axis and, therefore, no change was expected in the external moments or forces at any of the joints. Thus, for the purposes of this study, the axial rotation can also be thought of as a shift in the attachments of the muscles that cross the knee and ankle joints.

### Knee rotators sensitivity analysis

Five muscles that control non-sagittal plane rotations at the knee were selected and their PCSA was doubled relative to the original conditions of the model, to effectively double their upper force bound. These were gracilis, plantaris, popliteus, sartorius and tensor fascia latae (TFL). The model was re-run using the original motion data for these modified bounds, keeping all other parameters constant.

### Data analysis

The total number of frames that the model was unable to solve for each trial was collated and their proportion of the overall number of frames for that trial was calculated. The number of unsolved frames for each rotation condition and for the double upper bound was then averaged across all ten subjects. The frames that did not solve were then removed from the results for the remainder of the data analysis.

The joint force (shear, tibiofemoral and patellofemoral) and muscle force data was then smoothed using a Woltring filter to enable better comparison between trials and subjects. Only the five muscles that were used in the knee rotators sensitivity analysis were also selected for processing in the kinematic sensitivity analysis.

The jump motion was divided into four phases based on the kinematics and load response from the force plate.

1. Initial counter movement.
2. Take-off.
3. Landing.
4. Recovery.

Phases 1 and 2, and 3 and 4 were divided by the points of deepest knee flexion and phases 2 and 3 were separated by the airborne part of the jump. The peak shear, tibiofemoral and patellofemoral joint contact forces were identified in each phase and for each subject for the ‘original’ jump and compared with the corresponding frames in the altered model trials. These forces were normalised against the subject’s bodyweight and averaged across all subjects. The area under the force curve for each muscle was then calculated for each phase/condition/subject, normalised against bodyweight and the length of the airborne period, and then averaged across all subjects.

Finally, a repeated measures analysis of variance (ANOVA) was performed to statistically compare the mean peak loads and the mean area under the muscle curves, for each phase, between the original jump and the modified modelling conditions in the kinematic sensitivity analysis. A Bonferroni post-hoc test was then applied when appropriate. Paired t-tests were used in a similar manner to examine the differences in the results between the regular and doubled upper muscle bound modelling conditions. The significance level was set at p < 0.05.

## Results

The statistical analysis performed in the kinematic and knee rotators sensitivity analysis was a comparison against the results produced using the original kinematic data and muscle bounds. All statistically significant changes quoted are, therefore, relative to this original baseline data.

### Kinematic sensitivity analysis

The number of frames that would solve using the model was found to generally increase with external (negative) rotation but decrease with internal (positive) rotation, as seen in Table 1, however, this was not found to be statistically significant.

**Table 1.** Number of unsolved frames in the model output for the kinematic sensitivity analysis, as a percentage of overall frames for the motion of each subject. External rotation is negative and internal rotation is positive.

Subject	−0.1 rad	−0.05 rad	Original	+0.05 rad	+0.1 rad
1	1.2	1.2	1.2	1.4	1.2
2	0.2	0.2	0.2	0.2	0.7
3	1.2	1.6	2.2	2.9	4.0
4	2.9	2.4	2.1	2.7	2.4
5	1.8	1.8	1.8	1.8	1.8
6	4.5	7.3	14	20	22
7	2.0	2.5	3.1	4.6	15
8	2.3	2.0	2.5	2.5	2.8
9	2.0	3.2	6.8	9.2	10
10	7.7	10	17	21	25
Mean	2.6	3.2	5.0	6.6	8.5

**Figure 1.**
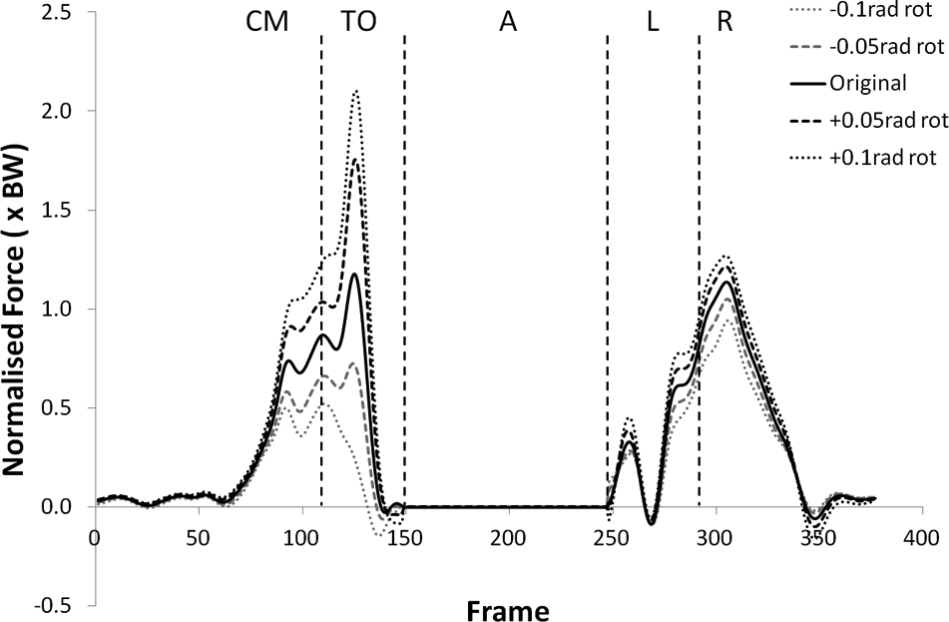
Shear force curves for subject 2 from the kinematic sensitivity analysis showing the variation with axial rotation of the shank segment. External rotation is negative and internal rotation is positive. CM: Counter Movement; TO: Take-Off; A: Airborne; L: Landing; R: Recovery phase.

**Figure 2.**
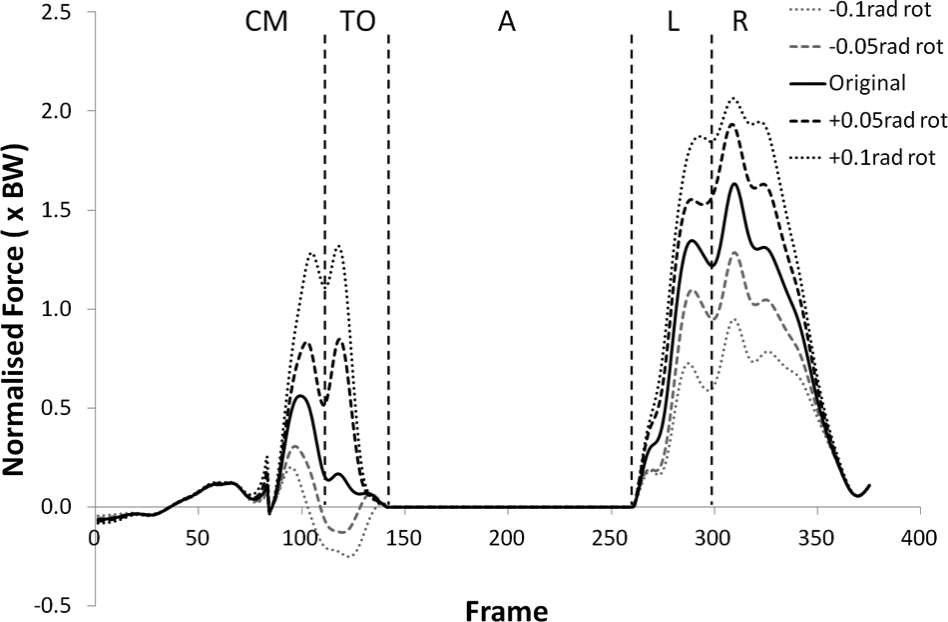
Shear force curves for subject 9 from the kinematic sensitivity analysis showing the variation with axial rotation of the shank segment. External rotation is negative and internal rotation is positive.

Many of the variables investigated displayed results for the modified kinematics that also followed the same profile as the original jump, but with scaled values during the regions of high loading. Figure 1 and Figure 2 provide examples of this and shows the shear force curves for subjects 2 and 9, which are typical of the results across most of the subjects. A summary of the statistical significance of the changes that occurred in the kinematic sensitivity analysis can be seen in Tables 2 and 3.

**Table 2.** Mean percentage changes in peak shear force, tibiofemoral joint contact force, patellofemoral joint contact force or area under the force curve (muscle forces) relative to the original kinematics, for the counter movement and take-off phases of the motion. External rotation is negative, internal rotation is positive and an asterisk indicates significant changes (p < 0.05)

Rotation angle (rads)	Counter movement				Take-off			
	−0.1	−0.05	0.05	0.1	−0.1	−0.05	0.05	0.1
Shear	−41	−21	35*	82*	−89	−51	78	180*
TFJ	−0.52	−0.71	−1.4	−1.7	1.1	0.62	−0.22	−1.5
PFJ	5.2	3.1	−3.5*	−5.3	5.0	3.1*	−2.6*	−5.0
Gracilis	4.1	2.0	−1.5	−2.1	4.1	−4.9	−5.3	−18
Plantaris	−17*	−9.3*	11	33	−13	−6.4	3.7	19
Popliteus	−33*	−16*	17*	32*	−25*	−11*	10*	27*
Sartorius	6.6	2.3	−1.5	0.46	91	43	−27	−42
TFL	−16	−9.1	9.3*	19*	−34*	−19*	20*	36*

TFJ: tibiofemoral joint; PFJ: patellofemoral joint; TFL: tensor fascia latae.

**Table 3.** Mean percentage changes in peak shear force, tibiofemoral joint contact force, patellofemoral joint contact force or area under the force curve (muscle forces) relative to the original kinematics, for the landing and recovery phases of the motion. External rotation is negative, internal rotation is positive and an asterisk indicates significant changes (p < 0.05)

Rotation angle (rads)	Landing				Recovery			
	−0.1	−0.05	0.05	0.1	−0.1	−0.05	0.05	0.1
Shear	−58	−26	30*	76	−58*	−29	24	75*
TFJ	0.34	0.21	−1	−0.77	1	0.28	1.8	1.5
PFJ	3.3	1.8	−2.3	−3.9	5.3	2.8	−2.2	−3.8
Gracilis	−8.4	−1.2	−6.5	−18	0.11	−0.39	−0.26	12
Plantaris	−21	−12	4.3	22	−8.7	−4.1	4.7	24.5
Popliteus	−36	−18*	16*	32*	−34*	−17*	17*	30*
Sartorius	39	19	−16	−33	32*	13	−7.1	−19
TFL	−30	−15	19	37	−27*	−14*	14*	28*

TFJ: tibiofemoral joint; PFJ: patellofemoral joint; TFL: tensor fascia latae.

**Figure 3.**
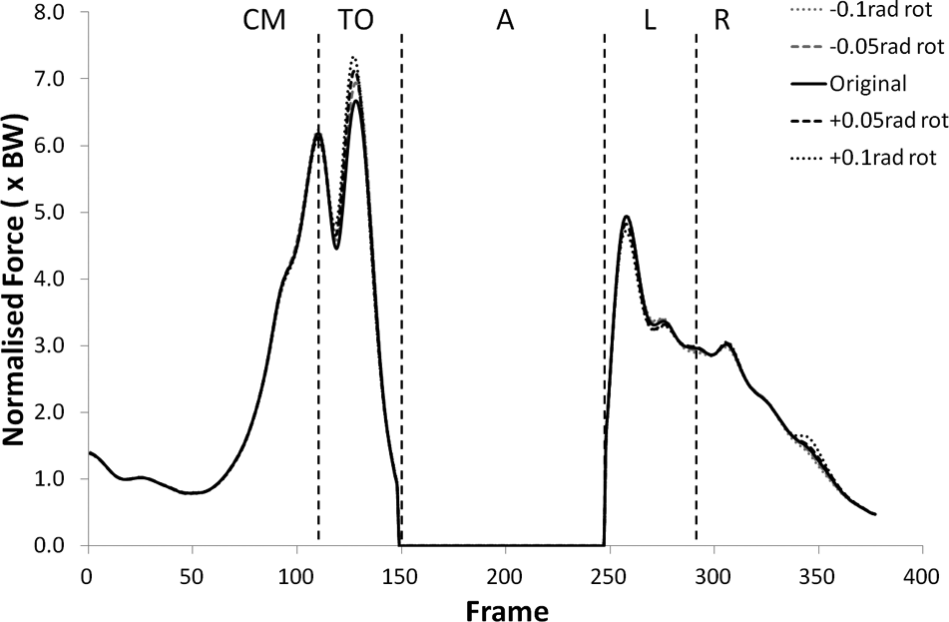
Tibiofemoral joint contact force curves for subject 2 from the kinematic sensitivity analysis showing the variation with axial rotation of the shank segment. External rotation is negative and internal rotation is positive.

**Figure 4.**
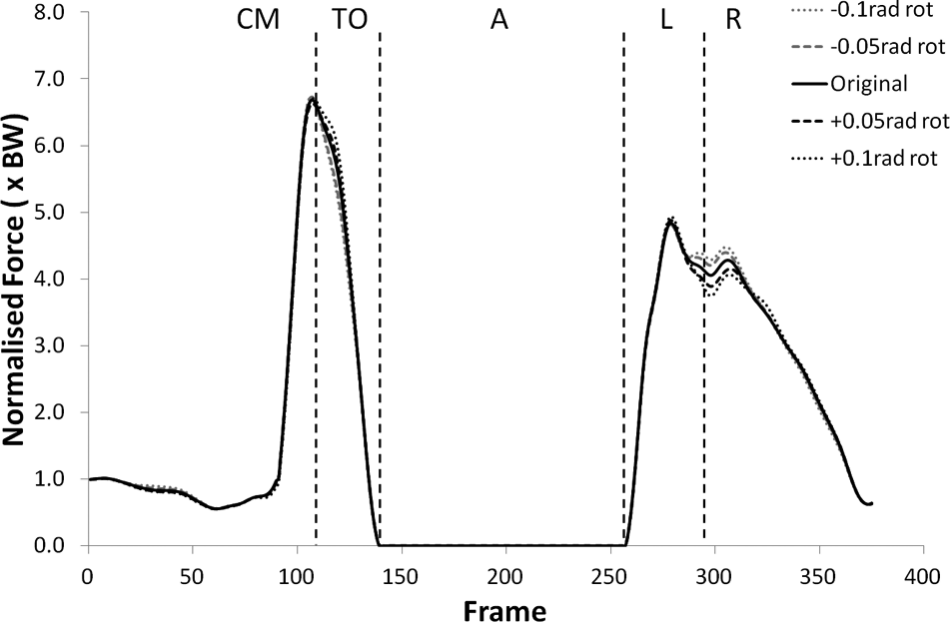
Tibiofemoral joint contact force curves for subject 9 from the kinematic sensitivity analysis showing the variation with axial rotation of the shank segment. External rotation is negative and internal rotation is positive.

**Figure 5.**
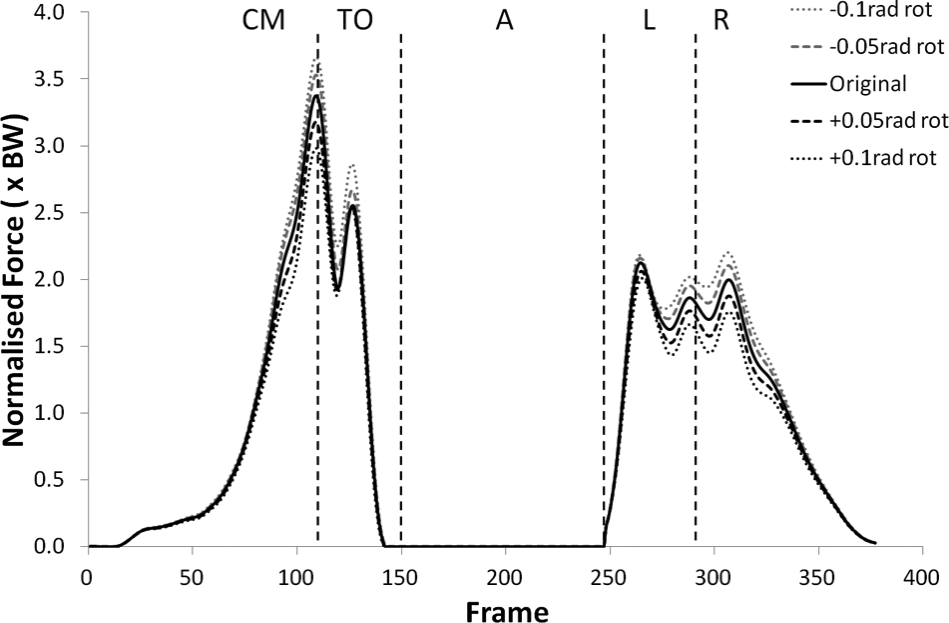
Patellofemoral joint contact force curves for subject 2 from the kinematic sensitivity analysis showing the variation with axial rotation of the shank segment. External rotation is negative and internal rotation is positive.

**Figure 6.**
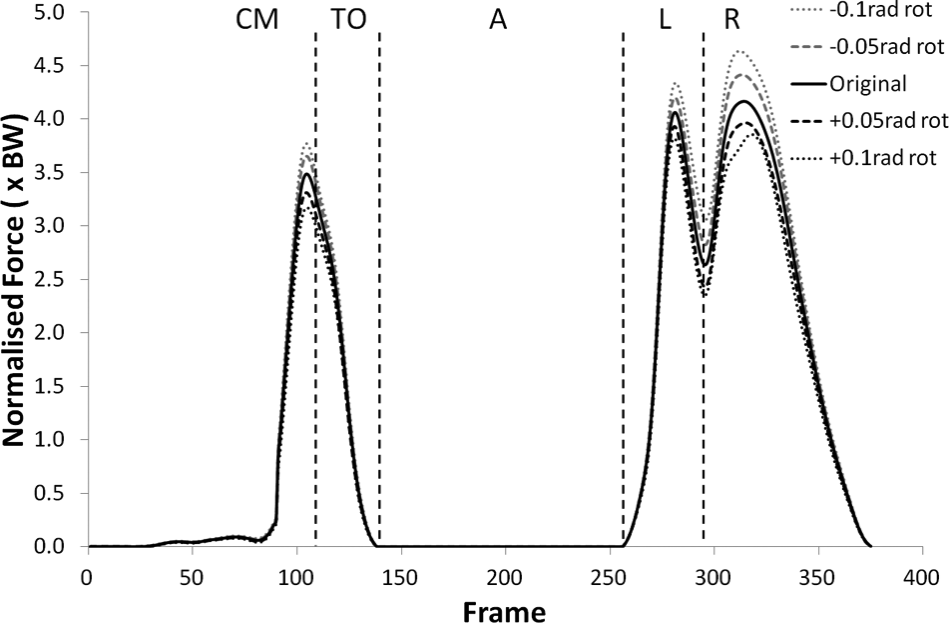
Patellofemoral joint contact force curves for subject 9 from the kinematic sensitivity analysis showing the variation with axial rotation of the shank segment. External rotation is negative and internal rotation is positive.

The peak values of shear force shifted negatively (i.e. more anterior) for external (negative) rotation, and shifted positively (more posterior) for internal (positive rotation). The shear force changes were statistically significant (p < 0.05) more often in internal rotation than external rotation except during the recovery phase. The peak tibiofemoral joint contact force (Figures 3 and 4) showed an increase with external rotation and decrease with internal rotation, but the changes were small and did not show statistical significance. This trend was more exaggerated in the peak patellofemoral joint contact force and was statistically significant during the counter movement (+0.05  rad, p < 0.05) and take-off (±0.05 rad, p < 0.05) phases (see Figures 5 and 6).

Two of the muscles, popliteus and TFL, showed a consistent decrease in force with external rotation and an increase with internal rotation. These trends were statistically significant for popliteus in all phases and rotations (p < 0.05) except −0.1 rad during landing, and for TFL in all phases and rotations (p < 0.05) except during counter movement (external rotation) and landing (all rotations). Plantaris showed similar trends but these were only statistically significant during the counter movement for external rotation (p < 0.05).

**Figure 7.**
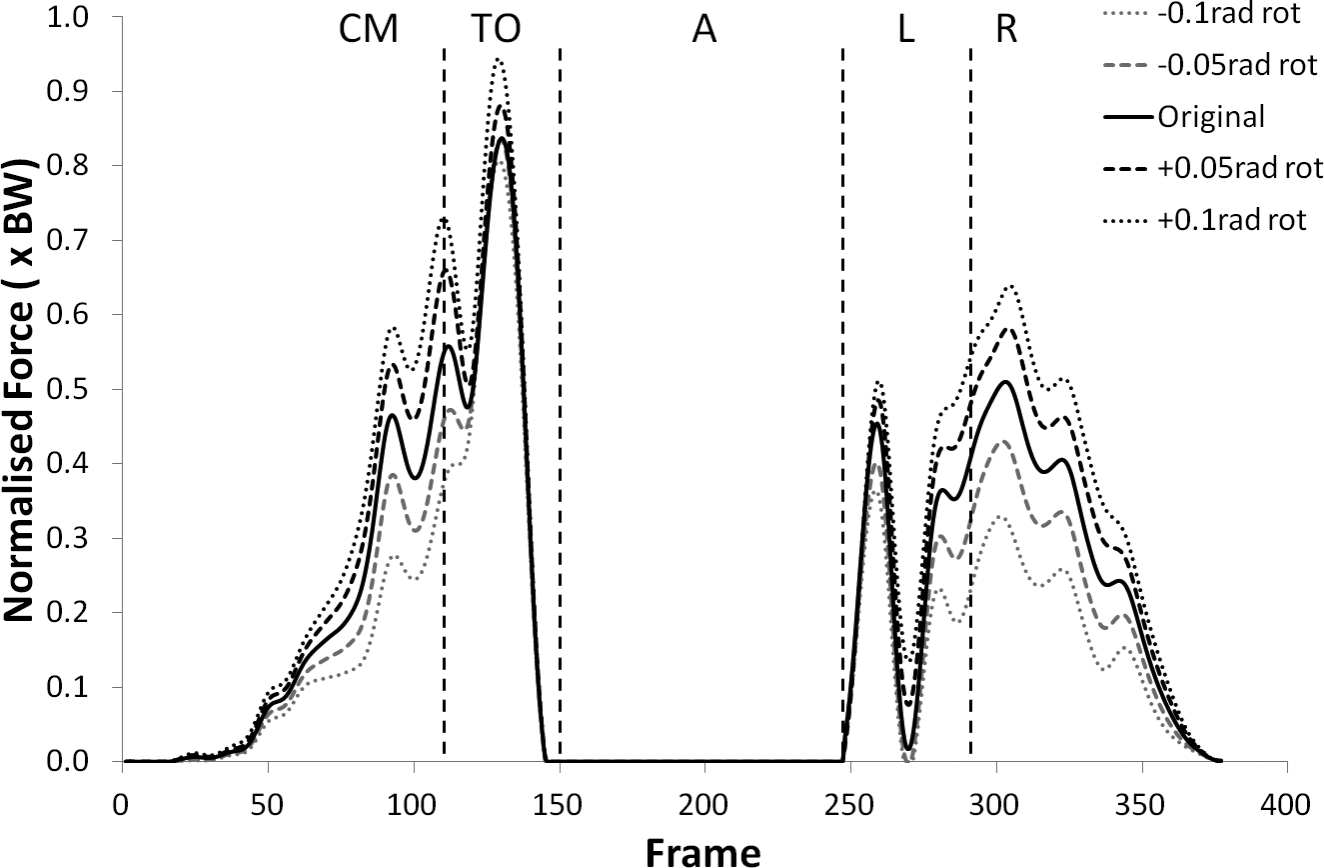
Subject 2 force curves for popliteus from the kinematic sensitivity analysis showing the variation with axial rotation of the shank segment. External rotation is negative and internal rotation is positive.

**Figure 8.**
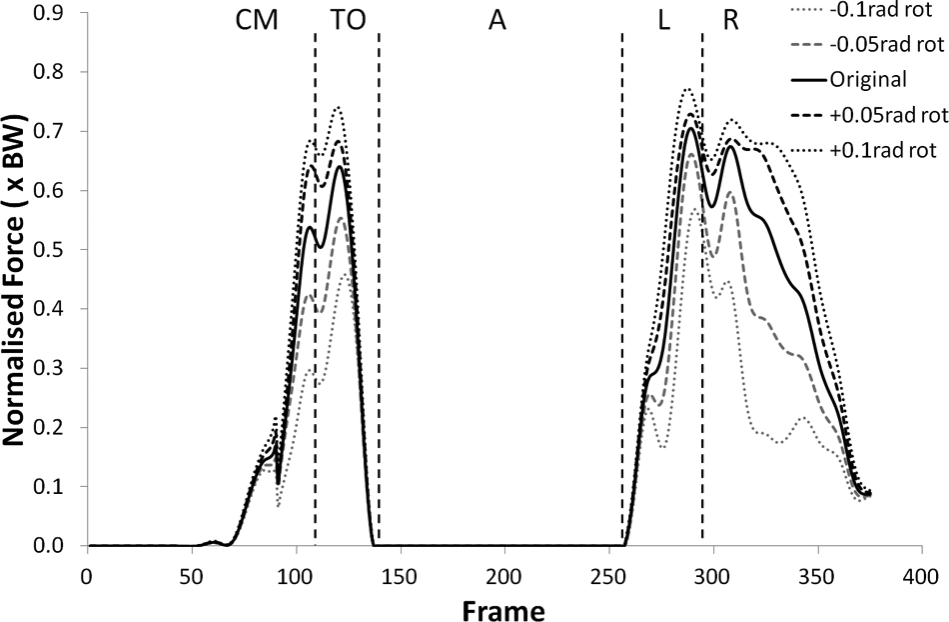
Subject 9 force curves for popliteus from the kinematic sensitivity analysis showing the variation with axial rotation of the shank segment. External rotation is negative and internal rotation is positive.

**Figure 9.**
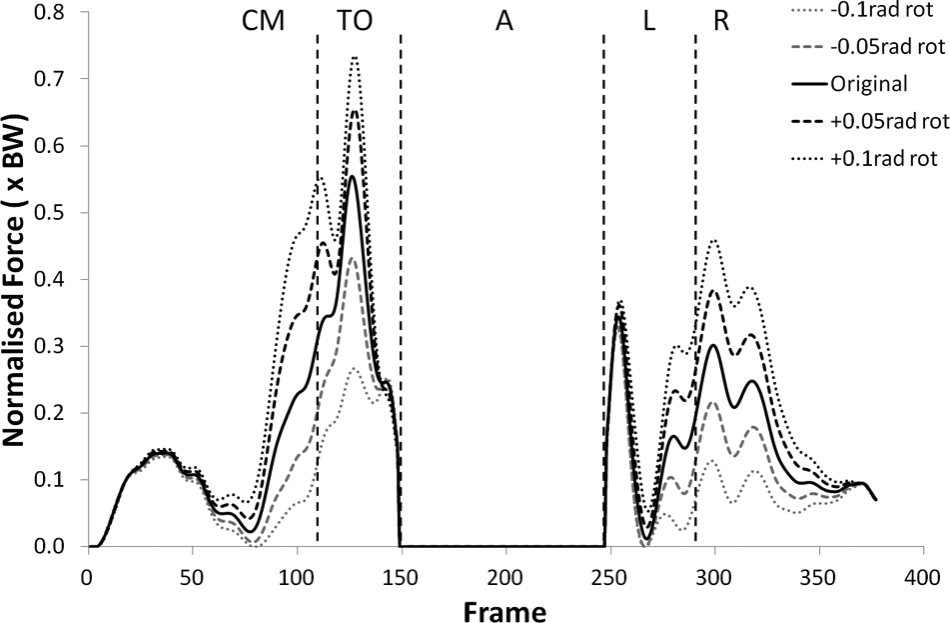
Subject 2 force curves for TFL from the kinematic sensitivity analysis showing the variation with axial rotation of the shank segment. External rotation is negative and internal rotation is positive.

**Figure 10.**
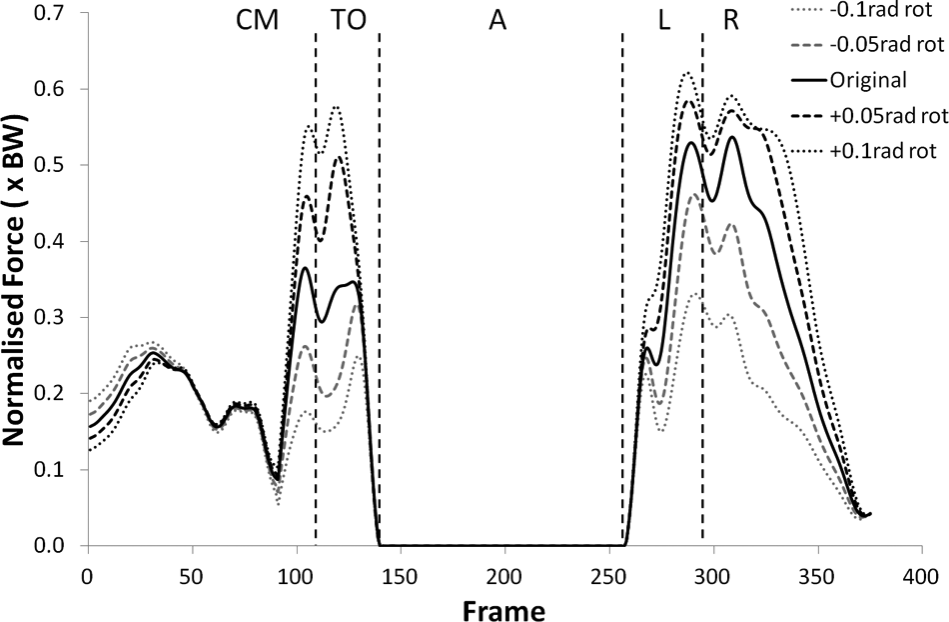
Subject 9 force curves for TFL from the kinematic sensitivity analysis showing the variation with axial rotation of the shank segment. External rotation is negative and internal rotation is positive.

Sartorius showed an increase in muscle force for external rotation and decrease for internal rotation, however, this was only statistically significant for −0.1 rad during recovery (p < 0.05). No clear trends were observed in the gracilis force over the range of rotations tested. The force curves for popliteus and TFL can be seen in Figures 7–10.

### Knee rotators sensitivity analysis

The number of frames that would solve using the model was found to significantly increase when doubling the upper bound of the knee rotator muscles (p < 0.05), as seen in Table 4. However, doubling the upper bound had less of an overall effect on the joint and muscle forces than altering the kinematics. Where changes did occur, they were again located at the regions of high loading. A summary of the statistical significance of the changes that occurred in the knee rotators sensitivity analysis can be seen in Table 5.

**Table 4.** Number of unsolved frames in the model output for the knee rotators sensitivity analysis, as a percentage of overall frames for the motion of each subject. An asterisk indicates a significant change from the original result (p<0.05).

Subject	Original	Double bound
1	1.2	1.2
2	0.20	0.20
3	2.2	0.52
4	2.1	1.6
5	1.8	1.8
6	14	2.8
7	3.1	1.3
8	2.5	1.6
9	6.8	1.2
10	17	3.4
Mean	5.0	1.6*

**Table 5.** Mean percentage changes in peak shear force, tibiofemoral joint contact force, patellofemoral joint contact force or area under the force curve (muscle forces) relative to the original kinematics, for each phase of the motion with the upper bound doubled for the knee rotator muscles. An asterisk indicates a significant change from the original results (p<0.05)

	Counter movement	Take-off	Landing	Recovery
Shear	−13	−21	−7.9	2.0
TFJ	1.6	2.6*	−0.94	−1.0
PFJ	3.1*	0.52*	2.4	2.3*
Gracilis	3.4	43*	6.5	43
Plantaris	47*	90*	88*	69*
Popliteus	−1.9	6.8	−4.3	−8.3*
Sartorius	3.2	45*	26*	14*
TFL	−3.5*	0.23	9.4	0.99

TFJ: tibiofemoral joint; PFJ: patellofemoral joint; TFL: tensor fascia latae.

**Figure 11.**
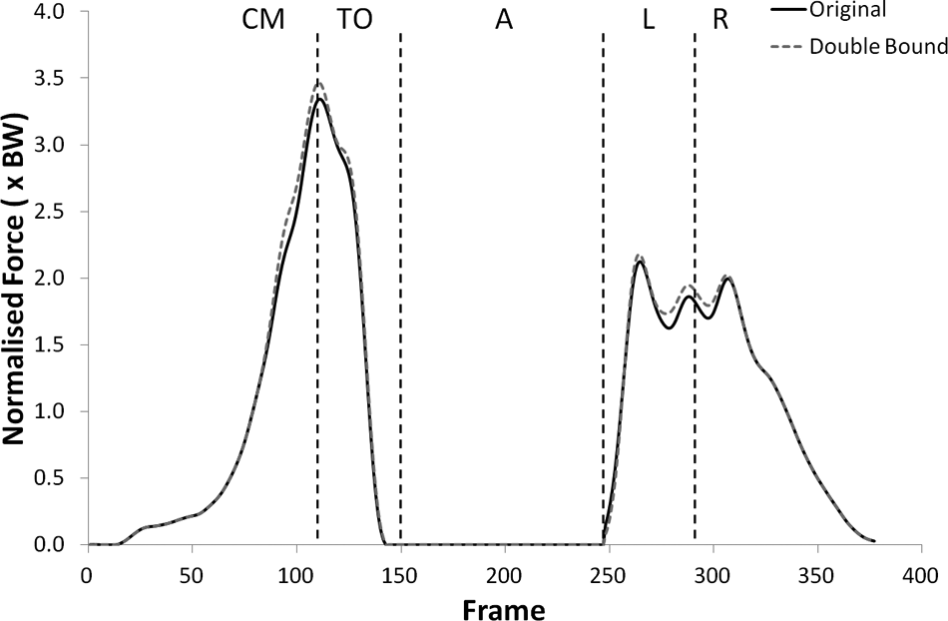
Patellofemoral joint contact force curves for subject 2 from the knee rotators sensitivity analysis showing the variation with the upper bound of the muscles doubled.

**Figure 12.**
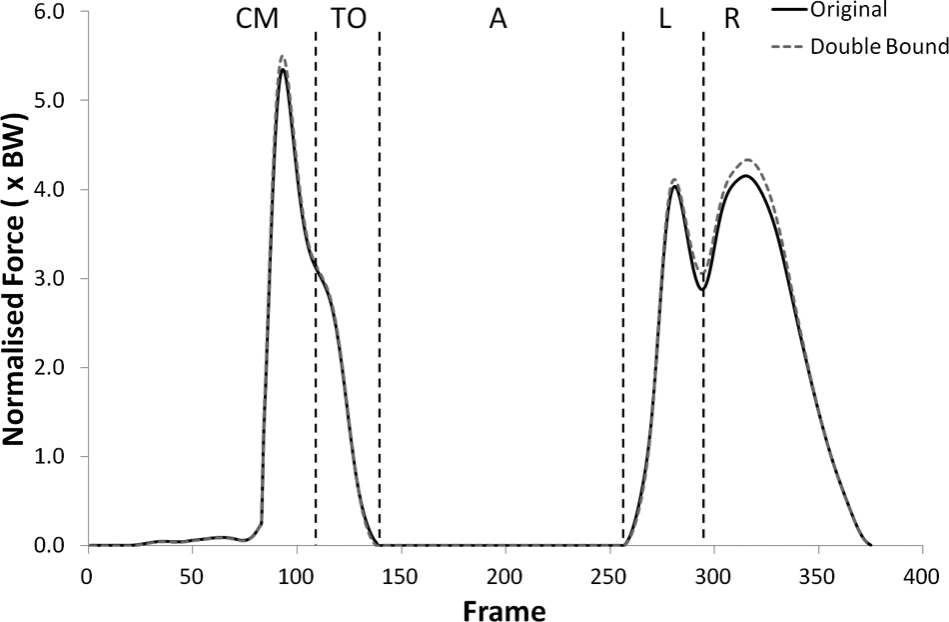
Patellofemoral joint contact force curves for subject 9 from the knee rotators sensitivity analysis showing the variation with the upper bound of the muscles doubled.

The shear force curves showed a trend towards lower forces with the double upper bound but no statistically significant changes were found. Significantly higher tibiofemoral joint contact forces were observed with the double upper bound during take-off (p < 0.05) only, but the patellofemoral joint contact forces were significantly higher during all phases but landing (see Figures 11 and 12, (p < 0.05)).

**Figure 13.**
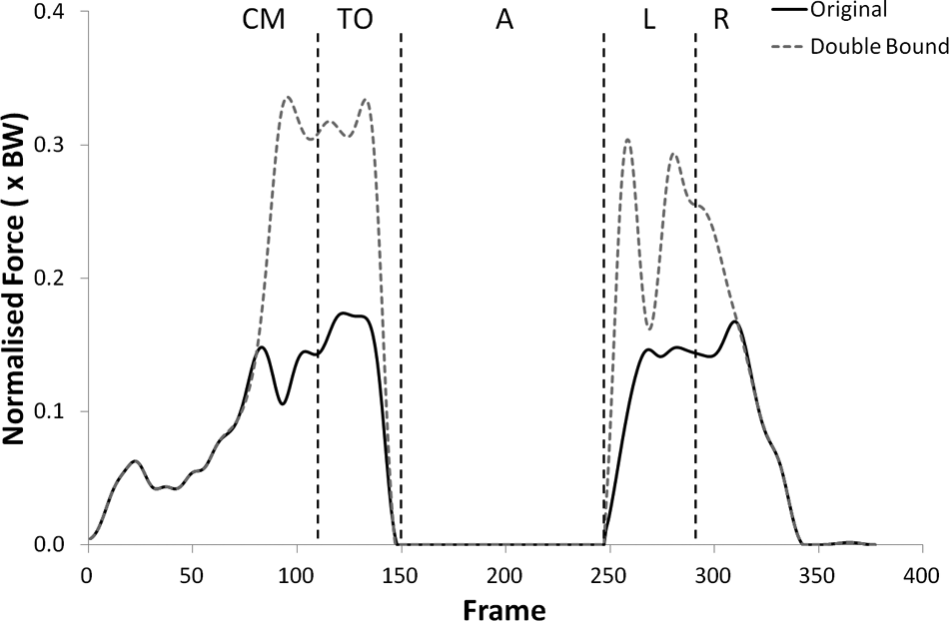
Subject 2 force curves for plantaris from the knee rotators sensitivity analysis showing the variation with the upper bound of the muscles doubled.

**Figure 14.**
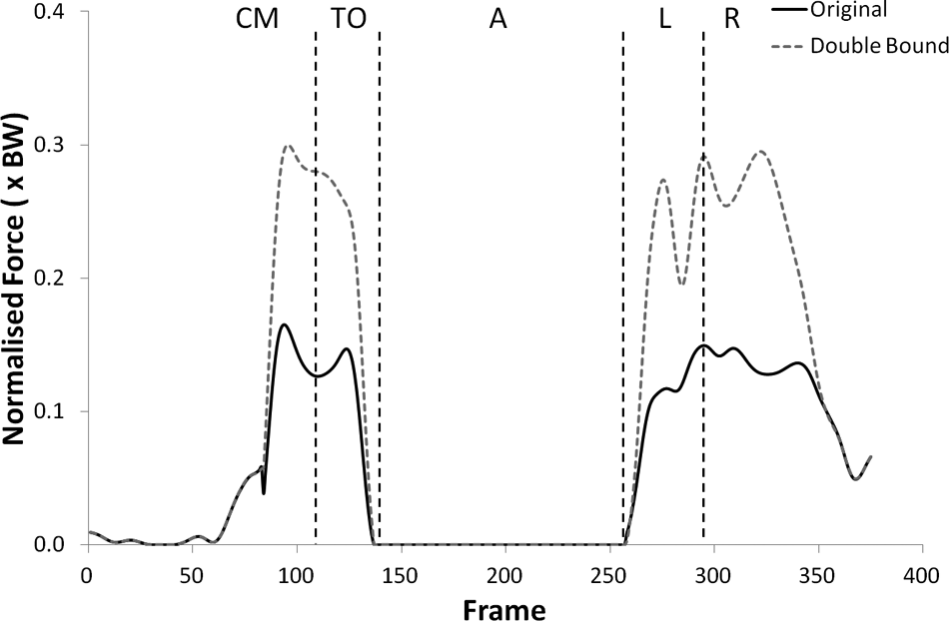
Subject 9 force curves for plantaris from the knee rotators sensitivity analysis showing the variation with the upper bound of the muscles doubled.

**Figure 15.**
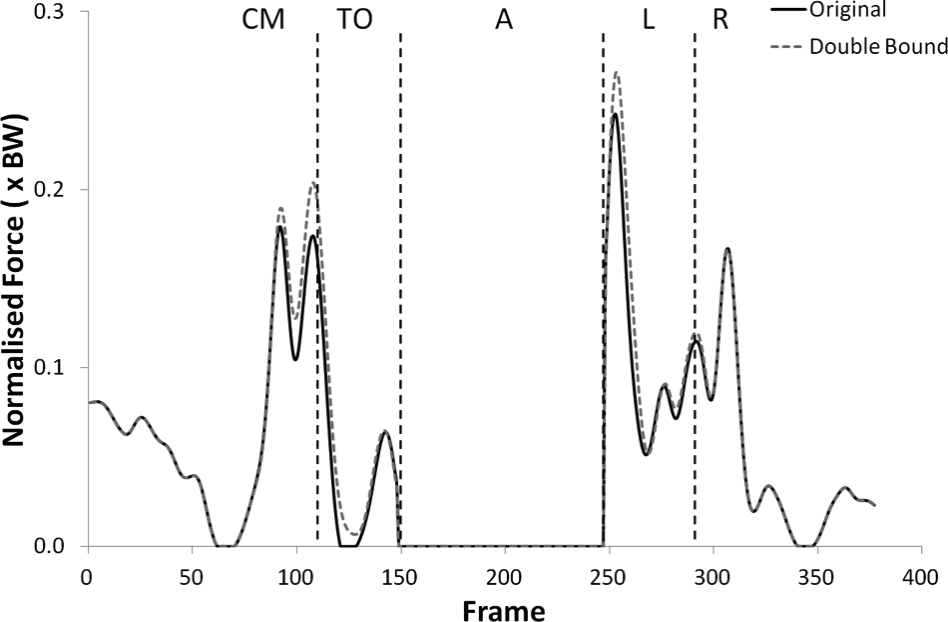
Subject 2 force curves for sartorius from the knee rotators sensitivity analysis showing the variation with the upper bound of the muscles doubled.

**Figure 16.**
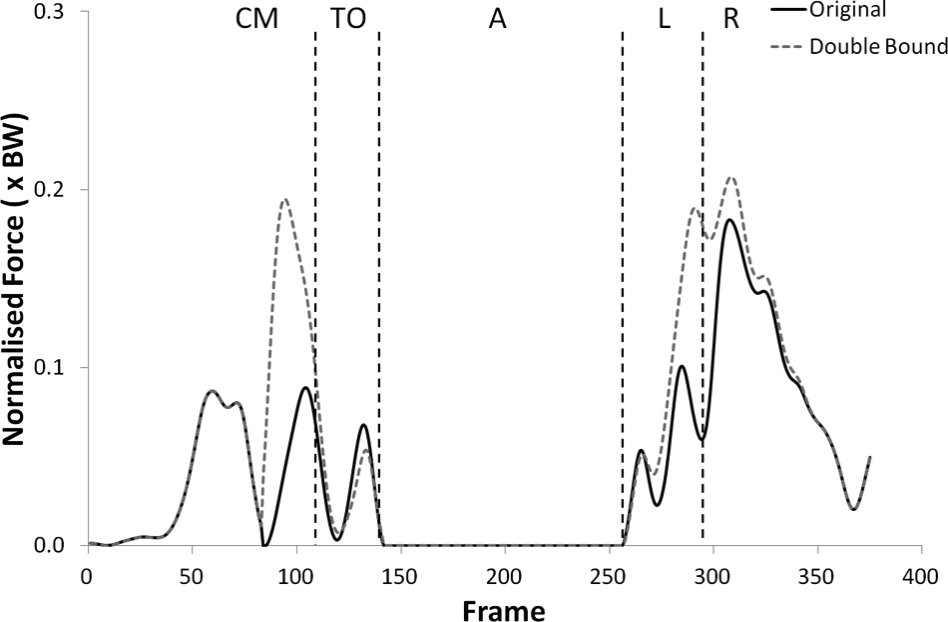
Subject 9 force curves for sartorius from the knee rotators sensitivity analysis showing the variation with the upper bound of the muscles doubled.

Higher muscle force levels were observed in plantaris and sartorius with the double upper bounds, as can be seen in Figures 13–16. These trends were significantly higher in all phases for plantaris and all but the counter movement for sartorius (p < 0.05). Gracilis also showed higher levels of force with the raised bounds, but it was only statistically significant during take-off (p < 0.05). Popliteus and TFL mostly displayed lower levels of muscle force but these were only statistically significant during recovery for popliteus and during the counter movement for TFL (p < 0.05).

## Discussion

The aim of this study was to investigate the effect of shank segment internal/external rotation and knee rotator muscle bounds on the joint reaction and muscle forces at the knee, as well as the ability of the model to find a valid solution for each frame. Clear trends were observed for the muscle and joint forces when altering the kinematics which showed that even moderate errors in marker position could have substantial effects on model outputs. Doubling the muscle bounds did not have as pronounced an effect on the muscle and joint forces, but it did have a significant impact on the ability of the model to solve for frames in highly loaded regions.

The increased ability of the model to solve during external rotation of the shank was accompanied by a decrease in muscle force for three of the muscles investigated; plantaris, popliteus and TFL. A closer inspection of the intermediate model outputs showed that, with external rotation, the moment arm of popliteus was increased about the superoinferior axis and the moment arm of TFL was increased about the anteroposterior axis. These are the main functional axes about which the muscles provide motion and the decrease in muscle force was most likely because the level of force required for the same moment production was reduced. Plantaris, however, does not have any origins or insertions on the shank segment and so any reduction in force must come as a result of changes in other muscles.

The shear force at the knee was found to move anteriorly with external rotation, which was an interesting finding as it coincided with increases in patellofemoral joint contact force, which in turn correlates with the patellar tendon force. A higher patellar tendon force would normally be associated with higher anterior shear forces, in knee flexion up to 90°. The peak muscle loads were generally found with the knee in deep flexion and in this position sartorius and gracilis could provide an opposing posterior force at the knee. However, little change was noted in gracilis and the elevated sartorius force may be balanced by the increase in popliteus force. This is a complex interaction that requires further analysis of the relative effect of the muscle forces on the shear loads at the knee.

While the two possible sources of kinematic errors have been mentioned (skin motion artifacts and incorrect landmark digitisation), it is also possible that rotational offset errors could arise from attempting to fit subject-specific marker data to generic skeletal geometry. In this case, it might not be that the segment as a whole is rotated, but that the muscle insertions on the bone are displaced slightly, however, from the point of view of the kinematic sensitivity study, it is a similar effect. Another result of incorrectly matched geometry might be that the moment arms of the muscles are altered, which would ultimately affect their moment-generating capacity. To this end, adjusting the knee rotator muscle upper bounds is also an investigation of the sensitivity of the model to this issue.

Relatively large differences in muscle force were observed for plantaris and sartorius with the double upper bound, showing significant increases in most phases of the motion. Increasing the PCSA reduces the stress for a given level of output force in a muscle and because the optimisation cost function aims to minimise the sum of the muscle stresses, this can cause the muscle recruitment strategy to change. Inspection of the remaining muscle forces showed that there was an accompanying reduction in soleus force which suggests that plantaris was selected preferentially to plantar flex the ankle. There was also a decrease in semitendinosus force, which could balance the increase in sartorius and maintain stability at the knee. The patellofemoral joint contact force was found to increase with the double upper bounds and this may also be owing to the elevated force in sartorius and plantaris. Both of these muscles’ lines of action lie on the posterior side of the joint and although their primary role is not as flexors, their increased muscle force would need to be balanced by higher force in the patellar tendon.

These observations represent good examples of the interaction effects that occur in musculoskeletal models that seek to equilibrate moments in 3D.^19^ The model did not feature a constrained knee axis, instead the knee joint centre was defined by the instantaneous orientation of the thigh and shank segments. The result of this is that the out-of-plane moments become part of the muscle force-sharing optimisation and, therefore, muscles are recruited to resist them. In single-axis knee models it is often argued that these moments are resisted purely by the ligaments. However, there are certain muscles, such as popliteus, whose line of action is not suited to motion about that axis, and to constrain the knee in this way prevents an investigation of its true function. Ultimately there is a use for both types of knee model and one that integrates both methods using a realistic ligament model will form part of the continuation of this study in the future.

It is difficult to be certain of the clinical implications of these sensitivities but the results seem to suggest that small changes in the alignment of the tibia relative to the femur could cause substantial changes in the shear loads at the knee. Obviously there are a number of structures in the joint that have not been modelled here and maybe translation of the joint can counteract some of the effects (such as changes in moment arms) to a certain extent. However, with further work perhaps, the results could be used to guide rehabilitation strategies so that targeted strengthening of the rotator muscles investigated in this study might reduce the risk of detrimental forces in the knee.

Increased anterior shear force is likely to generate higher loading in the anterior cruciate ligament (ACL) and, therefore, it may be that external rotation can put this structure at greater risk of injury, solely owing to the muscle forces that are required to equilibrate the moments at the joint. This would also increase translations of the tibiofemoral joint *in-vivo*, and in an ACL-deficient knee this may cause areas of cartilage to be loaded that would not normally be subject to load and may have further detrimental effects. It is possible that, in this scenario, the hamstring muscles may see greater levels of activation to restrict joint translation. However, the model was not constructed in a way that would require the muscles to balance the shear forces in the joint and so this was not an interaction that could be investigated.

Aside from the limitations inherent to musculoskeletal modelling, such as applying a generic model template to individual subjects, there were some specific limitations to this study. Only ten subjects were used in the sensitivity analyses and there was a certain degree of variability in the recorded motion data owing to slight differences in technique. Therefore, it was not possible to truly break the motion into a cycle with phases and average across subjects, as is normally conducted for gait. Areas of high loading were investigated as it was expected that any changes would be amplified in these regions, but it may be that this resulted in the omission of interesting features in other regions of the motion.

Passive-elastic ligament loads were not included in the version of the model used for this study to enable clearer comparisons of muscle forces without the added complexity of ligament interactions. The study was also not intended to be a comprehensive sensitivity analysis of the model, but instead for five muscles acting at the knee, axial rotation of a single segment and subsequent analysis of only one joint. The primary motivation was to explore the sensitivities to non-sagittal plane rotations that are often neglected or constrained to simplify models, but which may be detrimental to the accuracy of the results.^19^

There is a lack of motion analysis studies on this type of activity in the literature and to the best of the authors’ knowledge, none that have used electromyography (EMG) to assess the activation of the muscles investigated in this article. It was, therefore, not possible to compare the results of the sensitivity analysis with *in-vivo* estimations of the muscle activation. For this reason, and as with many modelling studies, the trends in the muscle and joint forces are of greater importance than the absolute values. However, an investigation using EMG methods or *in-vivo* measurements of forces for this activity would be extremely useful area for future study.

In conclusion, this study has provided some insight into the behaviour of a musculoskeletal model of the lower limb and has shown that rotational errors could lead to significant differences in predicted muscle and joint forces. Specific rotator muscles of the knee may also play an important role in balancing the out-of-plane moments and insufficient bounds may prevent a musculoskeletal model solving for high intensity activities.
